# Rupture of the Heart

**Published:** 1844-11-16

**Authors:** J. Howard Norton


RUPTURE OF THE HEART.

BY J. HOWARD NORTON, M. D., &C.

I was called, on the night of Oct. the 6th, to see a man, named Charles Blackman, carpenter, who had fallen down, apparently in a fit, and was dead before I could reach him. The account that his wife and a fellow-workman gave me of his previous state of health, was, that he had several times complained of a severe pain in the anterior portion, and at the left side, of the chest, but always continued to work at his trade up to the time of his death, and was considered a strong and active workman. The day previous to his decease, he had carried his carpenter's bench a short distance, and after having done so, complained to a fellow-workman "that he felt something inside him to have given way." On the following evening, he was seized, whilst in bed, with an acute pain in the chest, which he described as being very intense; he however walked down stairs to the yard, and almost instantly expired.

At the post-mortem, ordered by the coroner, I found, on the left side of the chest, extensive and old adhesions between the pulmonic and costal pleuræ (he having had several ribs fractured many years ago); both lungs were otherwise healthy. On opening the pericardium, I found it *filled* with dark coagulated blood; on examining the heart, I found a rupture, one quarter of an inch in length, at the superior part of the *right* ventricle, communicating with the cavity of the pericardium. The heart was enlarged without thickening of its walls; it was softer than natural; externally it presented signs of chronic inflammation; there were no pseudo membranes, or fibrinous adhesions; internally it had a pale and flabby appearance. On examining the surrounding parts, I found a common sewing needle, one inch and a half in length, imbedded in the cellular tissue attached to the anterior portion of the pericardium, but not piercing that membrane; this foreign body was evidently the exciting cause of the disease of the heart, which led to its rupture. No information could be obtained from his relations, as to how the needle had entered the body.—*Ibid.*

